# Pharmacy Technicians in Immunization Services: Mapping Roles and Responsibilities Through a Scoping Review

**DOI:** 10.3390/healthcare13151862

**Published:** 2025-07-30

**Authors:** Carolina Valeiro, Vítor Silva, Jorge Balteiro, Diane Patterson, Gilberto Bezerra, Karen Mealiff, Cristiano Matos, Ângelo Jesus, João Joaquim

**Affiliations:** 1 European Association of Pharmacy Technicians, B-1080 Brussels, Belgium; carolinavaleiro99@gmail.com (C.V.); president@eapt.info (C.M.); 2 Unidade Local de Saúde de Coimbra, EPE, 3004-561 Coimbra, Portugal; vahsilva@hotmail.com; 3 Escola Superior de Tecnologia da Saúde, Instituto Politécnico de Coimbra, 3046-854 Coimbra, Portugal; balteiro@estesc.ipc.pt; 4 Department of Pharmaceutical Sciences and Biotechnology, Technological University of the Shannon: Midlands Midwest, Dublin Road, N37 HD68 Athlone, Ireland; diane.patterson@tus.ie (D.P.); gilberto.bezerra@tus.ie (G.B.); karen.mealiff@tus.ie (K.M.); 5 LAQV/REQUIMTE, Escola Superior de Saúde, Instituto Politécnico do Porto, 4200-072 Porto, Portugal; acj@ess.ipp.pt

**Keywords:** pharmacy technician, immunization services, immunization, public health

## Abstract

**Background:** Pharmacy technicians are increasingly involved in immunization services, enhancing vaccine accessibility and reducing pharmacies’ workload. This scoping review aims to (1) provide a comprehensive overview of pharmacy technicians’ involvement in immunization services across various healthcare settings and countries, and (2) conduct a comparative analysis of training curricula for pharmacy technicians on immunization. **Methods**: A scoping review was conducted following the Arksey and O’Malley framework. A comprehensive search of the PubMed and Scopus databases was performed using keywords and MeSH terms such as “pharmacy technician(s)”, “immunization”, “vaccination”, “role”, and “involvement”. Studies included assessed pharmacy technicians’ roles in vaccine administration, training, and public health outcomes. Descriptive and thematic analyses were used to synthesize the findings. In addition, a supplementary analysis of immunization training curricula was conducted, reviewing programs from different countries to identify similarities, differences, and gaps in course structure, content, and delivery formats. Lastly, a comprehensive toolkit was developed, offering guidelines intended to facilitate the implementation of immunization training programs. **Results**: A total of 35 articles met the inclusion criteria, primarily from the United States of America (n = 30), Canada (n = 2), Ethiopia (n = 1), Denmark (n = 1) and United Kingdom (n = 1). The findings indicate that pharmacy technicians contribute significantly to vaccine administration, patient education, and workflow optimization, particularly in community pharmacies. The COVID-19 pandemic accelerated their involvement in immunization programs. Key challenges include regulatory barriers, a lack of standardized training, and resistance from other healthcare professionals. Facilitators include legislative support (e.g., the PREP Act), structured training programs, and collaborative pharmacist–technician models. **Conclusions**: Pharmacy technicians can play a vital role in expanding immunization services, improving vaccine uptake, and reducing pharmacist workload. Addressing regulatory inconsistencies, enhancing training, and fostering interprofessional collaboration are crucial for their effective integration of immunization programs. Since immunization by pharmacy technicians is not yet allowed in many EU countries, this review will provide a foundational basis to address their potential to support the healthcare workforce and improve access to immunization services.

## 1. Introduction

Immunizations are heralded as one of modern medicine’s greatest achievements, credited with saving up to 2.5 million lives annually [1]. Despite their efficacy, vaccine hesitancy—defined as the reluctance or refusal to vaccinate despite vaccine availability—has been identified by the World Health Organization (WHO) as one of the top ten global health threats, leading to underutilization of immunization services [2]. Barriers such as public complacency, concerns and misconceptions about vaccine safety and efficacy, costs, accessibility challenges, and convenience hinder optimal immunization coverage [3]. Annually, thousands of lives are lost worldwide that can be easily prevented by vaccination [4]. Global immunization efforts have saved an estimated 154 million lives—or the equivalent of 6 lives every minute of every year—over the past 50 years. The vast majority of lives saved—101 million—were infants [5,6] Vaccination continues to play a vital role in safeguarding public health, especially among older adults. By the end of 2021, close to 90% of individuals aged 60 and over in the EU had completed their initial COVID-19 vaccination series, with most countries surpassing 75% coverage. However, the uptake of booster doses showed significant variation. In early 2022, the rate of first booster uptake differed by a factor of seven between countries, and the disparity was even more pronounced for the second booster. While nations like Ireland and Denmark achieved coverage above 75%, others, such as Bulgaria, Romania, the Slovak Republic, and Lithuania, reported rates below 5%. For influenza vaccines, coverage initially increased during the first year of the pandemic but declined again in 2021–2022, though it remained higher than in pre-pandemic years. Persistent challenges such as vaccine hesitancy and limited access continue to hinder progress, with public confidence in vaccine safety differing markedly across the EU, ranging from 94% to just 60% [7].

Community pharmacies are widely regarded as one of the most accessible points of contact within the healthcare system, often serving as dependable sources of health information. Their convenient locations and extended hours, particularly in rural areas, make them essential in addressing individuals’ everyday medical needs [8]. Compared to other settings, such as clinics or hospitals, pharmacies offer greater convenience by providing vaccination services during extended hours, including evenings, weekends, and holidays [4]. Pharmacy-based immunization (PBI) has increased the vaccination rate [9]. PBI services have contributed to an estimated 6.2 million additional influenza immunizations annually between the years 2013–2018 [9] Moreover, a longitudinal study demonstrated a consistent rise in immunization rates through pharmacy-based programs, with coverage increasing from 75% in 2020 to 88% in 2022, highlighting their critical role in expanding vaccine access over time [9].

Over the past 15 years, different PBI strategies have emerged, leveraging pharmacies’ potential as centers for vaccine dispensing and immunization services [10] The extent of pharmacies’ involvement in immunization efforts has varied significantly across countries, reflecting differences in national policies, regulatory frameworks, and healthcare system integration [10,11]. This variation highlights the ongoing lack of consensus regarding the optimal role of pharmacies in vaccination programs, as well as a gap between the evidence supporting pharmacy-based immunization and its practical implementation [10] However, the growing need for immunization services demanded the creation of new opportunities and expanded the role of pharmacy professionals, including pharmacists and pharmacy technicians (PTs) [10]

PTs are qualified healthcare professionals who play a central role in supporting pharmacy services across multiple healthcare settings. They assist pharmacists in delivering essential pharmaceutical services, performing technical, operational, and increasingly clinical tasks that ensure the safe, effective, and efficient use of medications. PTs contribute to medication dispensing, health promotion, preparation of pharmaceutical formulations, inventory management, and the operation of complex technological systems. Their evolving role also includes participation in immunization programs, clinical trials, pharmaceutical industry activities, and regulatory agencies, with an increasing number of examples emerging globally. Their educational requirements, however, vary significantly across countries. Globally, the educational pathways for PTs range from secondary or upper secondary education to higher education, with formal programs typically lasting between 2 and 4 years, provided either through vocational education systems or higher education programs. This diversity in training reflects the lack of international harmonization regarding PTs’ education and scope of practice. These differences result in variability in competencies, roles, and legal authorizations for PTs, including their involvement in advanced pharmaceutical services such as immunization programs [12,13,14].

One of the major barriers to PBI is the absence of clear and supportive policies that formally recognize and authorize pharmacies as vaccination providers [15]. This lack of regulatory validation hinders the consistent integration of pharmacies into national immunization strategies, limiting their potential to expand vaccine access, particularly in underserved or hard-to-reach communities [16]. Even with specific regulations, the legal authority to administer vaccines varies across countries [16,17]. In different countries, like Portugal and the United Kingdom (UK), pharmacists are authorized to administer a range of vaccines, while others, like Belgium and the Netherlands, have more restrictive regulations, limiting the age of patients, only allowing particular vaccines (like Influenza and COVID-19 vaccines) [16,17]. These limitations and the lack of consensus highlight the need for national policies, including legislation, that recognizes PTs as authorized immunizers and establishes clear policies to support and validate the training of pharmacy professionals.

While pharmacists are frequently included as recognized immunizers in many countries, PTs are often disregarded, despite their potential to significantly contribute to expanding immunization capacity [18,19]. There is a growing interest globally in expanding the role of PTs within the immunization workforce, recognizing their potential to support vaccine delivery, increase operational efficiency, and enhance access, particularly in high-demand settings with limited healthcare personnel [18]. The involvement of pharmacy technicians in immunization services is a relatively recent development, with the earliest reported examples emerging in the United States following legislative changes in different states [20]. Their participation expanded significantly during the COVID-19 pandemic, which authorized PT participation in vaccination campaigns. Overall, the experiences reported in the literature have been largely positive, highlighting improvements in workflow efficiency, expanded vaccination coverage, and enhanced pharmacy service capacity.

According to the a recent FIP report (2025), 14 countries currently allow PTs to administer vaccines [21]. A cross-sectional study to evaluate the attitudes and experiences associated with pharmacy technician-administered immunization showed that pharmacists supported the statement that PTs could safely administer vaccines (>96% of respondents), and that the inclusion of PTs in vaccination improved the workflow of vaccine services (>82% of respondents) [22]. PTs have demonstrated effectiveness in administering immunizations in practice, with supervising pharmacists viewing these PTs as valuable members of the immunization team [9]. Furthermore, PTs reported increased job satisfaction and expressed a desire to take on a more integral role within the pharmacy team, including vaccination processes.

PTs have proven to be important in reducing the workload of pharmacists, particularly during periods of crisis such as the COVID-19 pandemic, when healthcare systems faced unprecedented demand. Their involvement helped maintain continuity of care and allowed pharmacists to focus on more complex clinical responsibilities [23]. In parallel, there has been a growing global interest in expanding the role of PTs within the immunization workforce. As healthcare delivery models evolve, leveraging the full potential of the pharmacy workforce, including trained PTs, has become essential to improving access and efficiency across immunization services [18].

This scoping review aims to (1) provide a comprehensive overview of pharmacy technicians’ involvement in immunization services across various healthcare settings and countries, and (2) conduct a comparative analysis of training curricula for pharmacy technicians on immunization, as well as propose an implementation guidelines toolkit for this. This approach allows for a broader understanding of international practices: first, it aims to provide an international overview of pharmacy technicians’ involvement in immunization services. This global mapping will serve to establish a broad contextual foundation, illustrating how pharmacy technicians are already contributing to immunization efforts worldwide. Building on these steps, a comparative analysis of immunization training curricula for pharmacy technicians allows a contrast between the more advanced integration of pharmacy technicians observed internationally—particularly in countries like the USA—with the current European educational landscape, where pharmacy technicians’ involvement in immunization is still limited or nonexistent. By connecting the global experience with the European context, this review aims to identify educational gaps and opportunities, ultimately informing the development of a model curriculum and an implementation guidelines toolkit tailored to the European healthcare and educational systems, by first mapping global involvement.

## 2. Materials and Methods

### 2.1. Study Design

This scoping review intends to map the pharmacy technicians’ involvement in immunization services. The methodological approach was based on the Arksey and O’Malley framework and adheres to the PRISMA-ScR (Preferred Reporting Items for Systematic Reviews and Meta-Analyses extension for Scoping Reviews) guidelines to ensure methodological transparency and rigor in reporting [24].

### 2.2. Search Strategy

**Figure 1:**
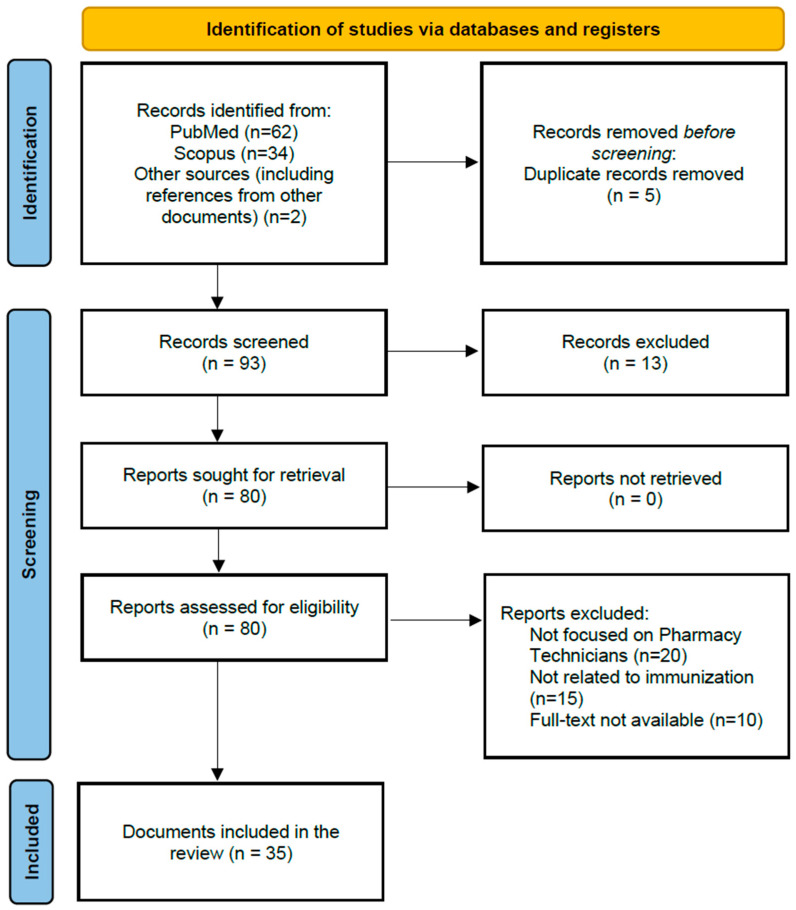
PRISMA flow chart diagram [24].

A literature search was conducted on PubMed and Scopus databases. Search terms included combination of free-text keywords and Medical Subject Headings (MeSH): “pharmacy technician(s),” “immunization,” “vaccination,” “role,” and “involvement”. The search was conducted without any time restrictions, allowing for the inclusion of all relevant sources regardless of their publication date. Gray literature sources and reference lists of key articles were also examined to ensure an inclusive review of the topic. Titles and abstracts were screened for relevance based on predefined eligibility criteria. Full-texts of potentially relevant articles were retrieved and assessed. Studies were included if they focused on pharmacy technicians’ involvement in immunization services, regardless of geographic location. The selection was conducted by two independent reviewers, with disagreements resolved by discussion. A total of 35 studies were included in the final review (Figure 1).

In addition to the literature review, a supplementary comparative analysis was conducted by C.V and V.S. to examine the structure and content of publicly available existing immunization training programs, to identify similarities, differences, and potential gaps. The findings were used to inform the development of a proposed educational model to support this professional competence.

The search included details on provider/organization, country, duration, format (online, hybrid, or on-site), and coverage of both theoretical and practical components.

### 2.3. Inclusion and Exclusion Criteria

The inclusion and exclusion criteria were established to ensure that only relevant studies were included in this review. Studies were eligible if they focused on pharmacy technicians’ roles in immunization services. Studies (1) involving PTs as a primary population, (2) examining their role in immunization/vaccination, and (3) employing any research design, including quantitative, qualitative, and mixed-methods approaches, were included in this review. No geographic restrictions were applied. Studies that did not include PTs, did not address immunization services, or were not available in English or not fully available were excluded.

For the website-based training program analysis, inclusion criteria consisted of (1) training programs targeted to pharmacy professionals, (2) programs with content related to immunization, and (3) sufficient detail available on the course content.

### 2.4. Data Charting and Extraction

A structured data extraction template was used to collect relevant information from each study. Extracted information included author(s), year of publication, type of study, country/region, objectives, pharmacy technician roles, vaccination type, and requested education/training (Table 1).

For the course content review, data were charted on provider organization, region, course duration, format, theoretical modules, practical modules and certification. Missing or unspecified modules were noted to highlight potential gaps in training (Supplementary Materials Table S1).

### 2.5. Data Synthesis

The extracted data were analyzed using both descriptive and thematic analysis methods. Descriptive analysis involved summarizing key characteristics of the studies, such as geographic distribution, study design, and intervention types. Thematic analysis was employed to identify recurring themes, including PTs’ roles, their impact on immunization rates, barriers to their expanded involvement, potential facilitators, training requirements, and implications for policy and practice.

Similarly, training programs were synthesized to identify patterns and discrepancies across countries and formats. This synthesis helped reveal inconsistencies in the preparation of PTs for immunization roles, particularly in the inclusion of public health concepts and scientific foundations of vaccine development.

No formal quality appraisal of the included studies was conducted, as the primary objective of this scoping review was to map the existing literature and describe the range of evidence available, regardless of methodological quality.

## 3. Results

### 3.1. Overview of Included Studies

This review included a wide range of studies that explored aspects of PTs’ involvement in immunization services. A total of 35 articles were included in this review, whose characteristics are given in Table 1. Most studies were conducted in the USA (n = 30), but also in Canada (n = 2), Ethiopia (n = 1), Denmark (n = 1) and United Kingdom (n = 1).

Hursman et al., Miran et al., McKeirnan et al., DeMarco et al., DiMario et al., Adams et al., and Arkeksey et al. focused on the impact of pharmacy technician-administered immunizations on public health, while others examined training requirements, regulatory barriers, and workflow integration [18,19,43,44,45,50,53,54].

A subset of studies focused on pharmacy technician-administered vaccines during the COVID-19 pandemic, documenting their role in expanding vaccination capacity through increased workforce availability and operational support [45,46,53].

Others evaluated long-term immunization initiatives, such as the implementation of training programs for PTs and the effectiveness of state-specific regulatory changes in enabling PTs’ participation [20,25,27,28,30,35,37,38,50,54].

A range of immunization types administered by PTs, including influenza, pneumococcal, hepatitis B, herpes zoster, and COVID-19 vaccines was also highlighted [18,26,39,45,46,50].

Different articles focused on the facilitators and barriers to expanding PTs’ immunization roles, identifying key regulatory, logistical, and societal challenges [18,29,31,32,33,34,35,37,39,41,42,43,44,45,47,48,51,52].

### 3.2. Role of Pharmacy Technicians as Immunizers

PTs have increasingly taken on roles in immunization services, particularly in vaccine administration, patient education, and logistical support [18].

The growing role of PTs in vaccine administration [22,26,27,32] were documented by different authors: McKeirnan et al. (2018) conducted a training program evaluation in Idaho, where PTs legally administered immunizations for the first time in the USA, demonstrating increased confidence and competency after training [30]. Similarly, DiMario et al. (2022) highlighted that a majority of surveyed pharmacists and PTs believed PTs could safely administer vaccines and improve workflow efficiency [22]. Adams et al. (2018) discussed the successful implementation of PT-administered vaccines in Idaho, Rhode Island, and Utah, setting a precedent for other states [34].

PTs have also contributed to immunization efforts beyond direct administration [44,50]. Pattin et al. (2017) emphasized their role in patient education, documentation, billing, and vaccine advocacy, particularly in underserved communities [26]. Programs like “VaxChamp” (Hursman et al., 2025) further illustrate how PTs can serve as vaccine champions, actively engaging with patients to address vaccine hesitancy and encourage uptake [53]. Furthermore, Hill et al. (2017) demonstrated that PTs in hospital settings play a critical role in patient immunization screening and coordination, ensuring higher vaccine compliance rates among hospitalized patients [27]. The expected competencies and roles of pharmacy technicians in immunization services are highlighted in Table 2.

### 3.3. Impact on Public Health

PTs’ involvement in immunization services has significantly improved vaccine coverage and accessibility, particularly in underserved populations [50,53,60]. During the COVID-19 pandemic, PT-administered vaccines contributed substantially to mass vaccination efforts [23,45,48,61,62]. In the UK, PTs were included in the national protocol to expand COVID-19 and influenza vaccination; the Association of Pharmacy Technicians UK (APTUK) position statement on this was that “any rapid redeployment of pharmacy technicians must be considerate of the impact on the safety, health and wellbeing of the individual and the wider implications for the delivery of core pharmacy services” [22,53,61]. In the USA, federal authorization enabled PTs across different states to participate actively in mass vaccination efforts, contributing to expanded capacity in retail and hospital settings [45].

Several studies have explored the role of PTs in improving immunization accessibility [22,27,50,53]. DiMario et al. (2022) found that PT-administered vaccines streamlined pharmacy workflow and increased efficiency, leading to higher vaccine uptake [22]. This is reinforced by Miran et al. (2024), who observed that pharmacists working with PT-immunizers reported enhanced job satisfaction and workflow improvements, enabling a more effective use of clinical resources [50]. Hursman et al. (2025) reported that programs like “*VaxChamp*” facilitated patient engagement, improving immunization rates for pneumococcal and hepatitis B vaccines [53]. Additionally, Hill et al. (2017) demonstrated that hospital-based technician interventions significantly increased inpatient influenza vaccination rates [27].

Research by Pattin et al. (2017) highlighted the impact of PTs in addressing immunization disparities, particularly in minority and low-income populations [26]. Community pharmacies positioned in medically underserved areas benefit from these encounters since the PTs serve as vital forces in diminishing health disparities by providing healthcare access to disadvantaged populations. By leveraging PTs’ frequent patient interactions, pharmacies have been able to identify vaccine-eligible individuals, provide education, and improve overall immunization coverage [34].

The requirements for PTs to administer vaccines vary by country and jurisdiction. In the USA, the Public Readiness and Emergency Preparedness (PREP) Act allowed PTs to administer vaccines under pharmacist supervision, provided they completed an accredited training program and maintained certification in basic life support [22,45,47,48]. 

Formal training programs, such as those accredited by the Accreditation Council for Pharmacy Education (ACPE), have been developed to ensure PTs are adequately prepared to administer vaccines [19,28,35,37,47,50].

Additional training requirements have been implemented in various pharmacy settings. For example, Hill et al. (2017) reported that PTs in hospital-based immunization programs received specialized training on patient assessment, vaccine storage, and documentation procedures [27]. Similarly, Hursman et al. (2025) described a program where PTs were trained in vaccine administration but also in patient communication strategies to address vaccine hesitancy [53].

Despite these advancements, challenges remain in standardizing training requirements across different regions.

### 3.4. Immunization Training Programs

Ten distinct programs were analyzed (Supplementary Table S1). These courses varied by country of origin, duration, ranging from brief 2 h modules to week-long sessions, and were offered in different formats, including online, hybrid, and in-person. Theoretical coverage was highly heterogeneous. For instance, Bain et al. (2009) found that only about 38% of pharmacy schools include immunization education as part of their core curricula, leading to significant variation in the theoretical preparation of both pharmacists and PTs [63]. Adams et al. (2022) identified at least 11 different training programs for PTs, noting substantial differences in program length, content, and instructional methods [19]. McKeirnan et al. described a training program that includes modules on public health, vaccine types, and immunology, but also highlights that these topics are not consistently included across all training programs [30,32]. Most programs provided detailed instruction on the more commonly used vaccines and their routes of administration, immunization schedules, administration techniques, vaccine-preventable diseases, and legal considerations. However, some modules—such as “Patient Follow-Up”, “Anatomy and Physiology”, and “Communication Skills”—were not indicated as integrated components in the course plans.

Based on our analysis, different modules that are considered essential components, such as “Public Health Concepts”, “Vaccine Pharmacology” and “Vaccine Development Process”, were not explicitly included in the curricula of any of the reviewed courses, indicating an incomplete coverage of contents. These omissions highlight gaps in preparing pharmacy personnel to understand the broader context of immunization within public health systems and the scientific basis of vaccine development.

Based on this review, after analyzing similar programs, the authors recommend that training programs for PTs should include, at least, the following components:

1. Foundational knowledge modules: covering the basic concepts of immunology, anatomy, physiology, public health, and pharmacovigilance.
2. Vaccine-specific modules: routes of administration, commonly used vaccines, vaccine-preventable diseases, vaccine schedules, vaccine storage, handling, and disposal.
3. Clinical safety: contraindications, management of adverse reactions and emergency response concepts.
4. Professional role development: role of PT in immunization.
5. Communication skills, particularly for patient interaction, consent procedures, and managing vaccine hesitancy.
6. Practical experience, including hands-on injection practice (supervised).
7. Personal safety and protection: instruction on the correct use of Personal Protective Equipment (PPE).
8. Life-saving response skills such as Cardiopulmonary Resuscitation (CPR) and emergency response training.

This information is expanded into Supplementary Table S2, where authors present a recommendation for Training Immunization Course Curricula for PTs.

### 3.5. Facilitators and Opportunities

Several factors have facilitated the expansion of pharmacy technician-administered immunization programs. Regulatory changes, such as the PREP Act in the USA, have allowed PTs to administer vaccines under pharmacist supervision, significantly expanding immunization capacity [45]. The PREP Act (Public Readiness and Emergency Preparedness Act) is important for pharmacy technicians administering vaccines because it provides legal authorization and liability protection, enabling them to expand their role during public health emergencies [64].

A retrospective data analysis conducted by Miran et al. (2024) compared the number of vaccines administered during the years from 2019 to 2021 (during the COVID-19 pandemic, after the PREP Act allowed PTs to administer vaccines) in pharmacies with immunizing PTs vs. those without, showing a great mean increase in vaccination volume in the former compared to the latter [50]. Following the expiration of the PREP Act’s emergency provisions, the role of PTs in immunization has evolved. Fuschetto et al. (2023) assessed the Ohio pharmacists and PTs regarding PT-administered vaccines [47]. Results showed 65.9% (n = 365) of pharmacists and 76.2% (n = 675) of PTs agreed that trained PTs should be able to administer vaccines post the COVID-19 pandemic [47]. Due to the strong interest expressed by both pharmacy and healthcare workers in their roles, several states have moved toward permanently adopting these changes. As of December 2024, PTs are permanently permitted to administer vaccines in 47 states [65].

### 3.6. Barriers and Challenges

Despite the success of pharmacy technician-administered immunization programs, several barriers hinder widespread implementation. In addition to training-related barriers, restrictive national legislation and resistance from other healthcare professional groups constitute significant obstacles to expanding pharmacy technicians’ roles in immunization services. Regulatory constraints remain a significant challenge, as not all countries or states permit PTs to administer vaccines. Adams et al. (2018) highlighted that state-specific regulations in the USA limit PT involvement in some areas, slowing down the expansion of these services [34]. Furthermore, Pattin et al. (2017) noted that inconsistencies in PTs’ training programs across jurisdictions create additional obstacles [26]. Logistical challenges also affect the integration of PTs into immunization programs. Hill et al. (2017) found that workload concerns, inadequate reimbursement models, and vaccine storage limitations were key barriers in hospital settings [27]. Similarly, Hursman et al. (2025) emphasized that staffing shortages and lack of clear role delineation for PTs can reduce the efficiency of vaccine administration programs [53].

Societal barriers, such as public trust and pharmacist acceptance, further complicate the expansion of PT-administered immunizations. Adams et al. (2023) reported that some patients remain hesitant about receiving vaccines from PTs rather than pharmacists [45].

These factors often stem from concerns about professional boundaries, scope of practice, and patient safety, highlighting the need for clear regulatory frameworks and interprofessional collaboration to facilitate the successful integration of pharmacy technicians into vaccination programs.

### 3.7. Implementation Guidelines

**Figure 2:**
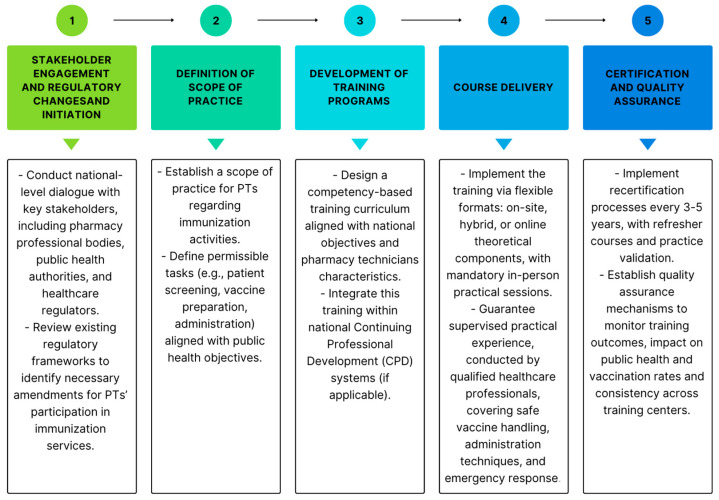
Implementation pathway for pharmacy technicians in immunization.

To support the formal integration of pharmacy technicians into immunization services, a set of structured guidelines for implementing immunization training program was developed. These recommendations aim to promote educational consistency, ensure competency-based training, and facilitate the safe and effective involvement of pharmacy technicians in vaccination practices (see Supplementary File S1 and Figure 2).

## 4. Discussion

The results illustrate the impact of PTs in immunization services, namely in administering vaccines, educating patients, and assisting in workflow processes [18,50]. Studies show that PTs played an important role in increasing vaccine coverage in poor socio-economic areas and mass vaccination campaigns during the COVID-19 pandemic [27,50,66]. Their participation helped increase the rates of influenza, pneumococcal, hepatitis B, herpes zoster, and COVID-19 vaccinations while lessening the burden on pharmacists [50]. Moreover, Miran et al. (2024) described an improvement in the workflow and vaccination rates, with PTs participating in immunization activities stating they feel more satisfied with their job (66.7%) [50]. Pharmacists felt that immunizing PTs positively impacted their job satisfaction (67.3%) [50].

However, some barriers prevent the full scope integration of PTs as immunizers, such as regulations, logistical barriers like workload and payment restrictions, and social issues like public perception and professional role clarity.

Several European countries have also begun to explore or implement expanded immunization roles to PTs within the community pharmacy settings; however, the published evidence is limited. In this review, there is a clear predominance of U.S.-based studies (30 out of 35), which reflects the current global distribution of published evidence regarding pharmacy technicians’ involvement in immunization services. Despite an extensive search of both peer-reviewed and gray literature, including documents from professional associations and public health agencies, published data from European countries remain very limited. This lack of European data is, in itself, an important finding, reinforcing the emerging nature of the subject within Europe. This geographic distribution also highlights the significant disparity in regulatory frameworks across countries and underscores the need for further research to better understand the role and potential impact of pharmacy technicians in immunization services within the European context.

The most recent International Pharmaceutical Federation (FIP) report (2025) further illustrates the limited European scope, with only Denmark, France, Norway, and the United Kingdom reporting pharmacy technician participation in immunization [21]. However, even in these countries, available information is scarce and fragmented. Moreover, these initiatives are recent: in France, PTs are allowed to administer all vaccines authorized for pharmacy use to individuals aged 11 and over. This expansion, effective from 6 December 2024, follows decrees published in the Official Journal, allowing PTs to vaccinate under the supervision of a pharmacist. Previously, their role was limited to administering influenza, COVID-19, and Mpox vaccines. The new regulations require PTs to undergo additional training to administer the full range of vaccines (Diphtheria, Tetanus, Poliomyelitis, Whooping Cough, Influenza, Human Papillomavirus, Measles, Mumps, Rubella, Hepatitis A, Hepatitis B, Meningococcal ACYW, Meningococcal B, Pneumococcus, Chickenpox, Shingles, Yellow Fever, Rabies) for individuals over 16 years old and prescribe vaccination renewals for the same 18 vaccines [67,68,69]. In England, PTs have been authorized to administer influenza vaccines under the national protocol since the 2021/2022 flu season. The administration of flu, COVID, and travel vaccinations under a Patient Group Direction (PGD) is already established practice in the UK [49]. Additionally, a pilot program on the Isle of Wight demonstrated the successful involvement of PTs in administering nasal flu vaccinations to children aged 4–11 years in school settings [70,71]. In 2015, Danish community pharmacies began offering travel vaccinations, and this service has since expanded to an increasing number of locations. The program also includes influenza vaccination and, most recently (in April 2020), pneumococcal vaccination; both are remunerated for defined risk groups [40]. In Denmark, community pharmacists and PTs can administer vaccinations, but only under the supervision of a physician. Danish law allows physicians to delegate certain healthcare tasks, like giving vaccines, to other professionals such as pharmacists, PTs, or nurses, as long as the responsibilities are clearly defined by both the law and the supervising physician [40].

Although PTs have been acknowledged for their contributions to the administration of vaccines and patient education, their participation in these roles still faces professional opposition and regulatory issues. DiMario et al. (2022) showed that while many PTs could appropriately administer vaccines and assist in workflow optimization, some pharmacists continue to express reservations about further expanding PTs’ roles [22]. In a survey performed by Kulczycki et al. (2021) to assess pharmacists’ views on the role of PTs in immunization, only 24% of pharmacists agreed that PTs should be allowed to administer vaccines, even though most believed that PTs should actively participate in the screening and evaluation of patients [72]. This situation shows that there is still a lack of advocacy and education concerning the implementation of pharmacy technician-administered immunization services [72].

Immunization programs managed through pharmacies have shown promise in expanding vaccine coverage [73]. In the USA and Canada, where PTs can administer vaccinations, some studies have reported improved access and enhanced workflow management [1]. These factors may, in turn, support higher immunization uptake; however, other factors, such as policy initiatives, legislation, and general healthcare accessibility, also play a significant role in influencing vaccination coverage. Furthermore, effective models like pharmacy-driven vaccination efforts in cystic fibrosis clinics show how including PTs in immunization services could improve patient outcomes as well as lower the demand placed on other healthcare professionals [74]. Moreover, handing over these bigger responsibilities to PTs increases job satisfaction [75]. Nonetheless, differences in national and local laws remain a challenge. A study from Latin America (Argentina, Brazil, Chile, Colombia, Mexico and Venezuela) on pharmacy practice noted that the absence of supportive policies, unsatisfactory training, and opposition from pharmacists were major hindrances to pharmacist-operated immunization services [76]. Moreover, the lack of funding, such as for educational opportunities and paying for vaccination services, has been recognized as an important barrier to greater use of vaccines administered by PTs [15].

The growth of pharmacy technician responsibilities, especially in vaccine services, led to better employee motivation and professional identification, and longer-term employment within the sector. Besides the Miran et al. study mentioned previously [50], McKeirnan & Hanson (2023) reported that PTs experience enhanced professional fulfillment and a sense of workplace affiliation following the chance to actively participate in clinical practice [44]. Health service optimization and physician retention result from transferring clinical work to PTs, thus helping stabilize this sector, where personnel frequently leave [44].

However, potential enabling factors have also been identified that may support the expanded uptake of pharmacy technician-administered immunizations, such as regulatory changes, professional advocacy, and demonstrated safety and efficiency [18,19].

Integrating technology such as digital health records, mobile applications, and virtual communication tools with structured and standardized immunization training programs can help PTs become more accountable to take on the further responsibilities of vaccine administration [77]. The benefits of immunization programs led by pharmacy technicians highlight the need to adapt pharmacy education to better prepare students for vaccine administration [78]. This shift can be made possible by coming up with national strategies that allow the training and certification of PTs to make them eligible for vaccine administration [79].

Research primarily examines pharmacist experiences about vaccination practices even though PTs increasingly participate in these procedures. Gerges et al. (2023) present a qualitative study focusing on PTs’ vaccine administration, yet this work only includes a short reference to their role, without exploring their perspectives in depth or providing a systematic assessment [62]. Urgent action needs to be taken to revise research and educational strategies by enhancing the active engagement of PTs throughout the design, implementation, and evaluation of studies and public health policy development, as well as in continuous training. Support mechanisms paired with competency evaluations and defined responsibilities should accompany PTs during their work with vaccinations to prevent work imbalances and maintain patient safety.

Despite the valuable insights gained from this scoping review, several limitations must be acknowledged. First, most of the included studies were conducted in the USA, with limited representation from other countries due to the early-stage development of pharmacy technician-administered immunization services in many regions, particularly in Europe, where regulatory frameworks and published research on the topic remain scarce. Additionally, the rapid expansion of PT roles during the COVID-19 pandemic was predominantly documented in U.S.-based literature, reflecting a more advanced integration of technicians in vaccination efforts in that context. Another limitation is the variability in study designs and methodologies. While some studies provided quantitative data on vaccination rates and workflow efficiency, others relied on qualitative assessments of perceptions and experiences. The lack of standardized outcome measures makes it challenging to directly compare findings across studies. Additionally, many studies focused on short-term interventions, particularly during the COVID-19 pandemic, without examining the ongoing feasibility and effectiveness of these programs in routine vaccination efforts.

Furthermore, it is relevant to acknowledge that a previous scoping review by DeMarco et al. (2022) addressed similar objectives, which included studies up to June 2020, representing a pre-COVID-19 perspective [18]. Since then, immunization policies and pharmacy practice models have evolved rapidly, especially in community pharmacy settings. In our scoping review, 18 out of the 35 included studies (51%) were published after 2020, highlighting a significant growth of evidence in the post-pandemic period and supporting the importance of this updated review.

The integration of PTs into vaccination services offers several key advantages: for PTs, it represents a clear opportunity for professional development by enhancing their clinical skillset, improving job satisfaction, and fostering a greater sense of contribution to public health. For patients, the benefits are equally compelling: improved access to timely vaccination, particularly in underserved or rural areas, shorter wait times, and more efficient pharmacy operations. In this way, the expanded role of PTs supports workforce optimization and contributes meaningfully to patient-centered healthcare delivery.

## 5. Conclusions

This scoping review highlights the expanding role of pharmacy technicians (PTs) in immunization services, demonstrating their growing contribution to vaccine administration, logistical support, and patient education. The available evidence indicates that integrating PTs into immunization services improves vaccination coverage, enhances workflow efficiency within pharmacies, and increases job satisfaction among pharmacy staff, particularly in high-demand settings such as during the COVID-19 pandemic.

However, several barriers continue to hinder the full integration of PTs as immunizers, including regulatory limitations, inconsistent training requirements, and varying levels of professional acceptance. The lack of harmonized policies and standardized educational pathways limits the potential of PTs to contribute more broadly, especially in Europe, where published evidence remains scarce and fragmented.

Our findings emphasize the need for clear national policies recognizing pharmacy technicians as competent immunizers, supported by structured, competency-based training programs.

## Figures and Tables

**Table 1:** Characteristics of included studies on pharmacy technicians’ involvement in immunization services and their main findings.

Author, Year	Type of Study	Country	Objective	Pharmacy Technician Role	Vaccination Type	Requested Education/Training
Powers & Hohmeier, 2011 [20]	Literature review	USA	Identify opportunities for PTs in immunization programs	Support roles such as documentation, billing, adverse event reporting	General immunizations	CPR training recommended, but no direct administration training
Rhodes et al., 2016 [25]	Interventional	USA (North Carolina)	Implement a vaccine screening program in an independent community pharmacy	Identified eligible patients for vaccination	Influenza, pneumococcal, Tdap, zoster	No direct immunization training; screening role only
Pattin, 2017 [26]	Literature review	USA	Review disparities in immunization uptake and PTs’ roles	Assisted in documentation, billing, patient education	General immunizations	No direct administration training, but involvement in vaccine workflow
Hill et al., 2017 [27]	Interventional (pre–post)	USA (Kansas)	Develop a PT-driven inpatient vaccination program	Identified and screened hospitalized patients for vaccination	Influenza, pneumococcal	Training on patient screening, communication, and documentation
Adams and Bright, 2017 [28]	Other (letter)	USA (Idaho)	Discuss PT-administered vaccines under new state law	Administered vaccines under pharmacist supervision	General immunizations	National certification, ACPE training, basic life support certification
Atkinson et al., 2017 [29]	Other (commentary)	USA	Discuss potential role of technicians in vaccine administration	Administered vaccines under pharmacist supervision	General immunizations	Training in administration techniques and basic life support
McKeirnan et al., 2018 [30]	Interventional (pre–post)	USA (Idaho)	Evaluate the effectiveness of an immunization training program for PTs	Administered vaccines after training	General immunizations	A 4 h training program (2 h home study, 2 h live training)
Westrick et al., 2018 [31]	Observational (cross-sectional)	USA	National survey on pharmacy-based immunization services	Technicians supported vaccine workflow but did not administer vaccines	Influenza, pneumococcal, herpes zoster, Tdap	No direct immunization training, but involved in administrative roles
McKeirnan et al., 2018 [32]	Literature review	USA	Describe and support the advancement of PTs roles in community pharmacy	Administered immunizations, performed final product verification and verbal communication, and managed medication synchronization	General immunization	ACPE Certificate for PTs, basic life support certification
Doucette & Schommer, 2018 [33]	Observational (cross-sectional)	USA	Assess PTs’ willingness to take on emerging tasks	Low willingness to administer vaccines	General immunizations	No specific training reported
Adams et al., 2018 [34]	Other (letter)	USA	Discuss perceptions and realities of pharmacy technician-administered vaccines	Administered vaccines under pharmacist supervision	General immunizations	State-dependent, some required formal training
Bertsch et al., 2019 [9]	Qualitative study	USA	Assess the perceptions of supervising pharmacists	Administering immunizations under pharmacist supervision	General immunization	ACPE-accredited immunization training and basic life support certification
Huston et al., 2019 [35]	Qualitative study	USA (Alabama, California)	Assess pharmacist perceptions of a community pharmacy immunization enhancement program	PTs supported workflow, identified high-risk patients	Pneumococcal, zoster	ACPE-accredited immunization training for pharmacists and PTs
Eid et al., 2019 [36]	Literature review (policy analysis)	USA	Review legal landscape of PT vaccine administration across all USA	Varied by state; three states (Idaho, Rhode Island, Utah) authorized administration	General immunizations	Varies by state, some requiring formal immunization training
Gavaza et al., 2020 [37]	Observational (cross-sectional)	USA (California)	Assess opinions of pharmacists and PTs on allowing PTs to administer immunizations	Not currently permitted; study explores support for future authorization	General immunizations	If authorized, training would be required (e.g., ACPE-accredited programs)
Bertsch & McKeirnan, 2020 [38]	Qualitative study	USA	Assess pharmacists’ perceptions of immunization-trained PTs	Administered vaccines, improved workflow	General immunizations	Limited training availability identified as a barrier
Burke, 2020 [39]	Other (opinion)	USA	Advocate for expanding PTs roles, including immunization administration	Potential future role in vaccine administration	General immunizations	Inconsistencies in training requirements noted as a barrier
Hansen et al., 2021 [40]	Other (commentary)	Denmark	Analyze the evolving role of Danish community pharmacies in public health following national strategies and legislative changes.	Handle vaccinations on the responsibility of a physician for a patient group, defined by law and by the physician	Travel, influenza, pneumococcal	Not provided
McKeirnan et al., 2021 [41]	Observational	USA	Assess patient perceptions and acceptance of PTs serving as immunizers	Administering immunizations under pharmacist supervision	General immunization	Certified pharmacy technicians with additional immunization training and basic life support certification
Sparkmon et al., 2021 [42]	Observational (cross-sectional)	USA	Determine predictors of pharmacists’ comfort levels with certified and trained technicians performing advanced functions.	PTs were evaluated in roles involving verbal prescription communication, nonclinical medication therapy management support, vaccine administration and prescription verification	General immunizations	Nationally certified and trained in the specific advanced task in question
Hohmeier et al., 2021 [43]	Other (commentary)	USA	Explore team-centered approach to immunization in pharmacies	Assisted in vaccine workflow, documentation, and patient interaction	Influenza, COVID-19	On-the-job training, some states required formal certification
Adams et al., 2022 [19]	Other (commentary)	USA	Review five years of PT-administered immunizations	Administered vaccines in various states	General immunizations	Multiple training programs available, including ACPE-approved courses
DiMario et al., 2022 [22]	Observational (cross-sectional)	USA	Compare pharmacists’ and PTs’ attitudes on technician-administered vaccines	Administered vaccines, handled billing and workflow	ACIP-recommended immunizations, COVID-19	PREP Act-authorized training and state regulations
DeMarco et al., 2022 [18]	Literature review	Canada	Review the role of PTs in vaccination services	Screening patients, administering vaccines	General immunizations	Advocacy for accredited vaccine administration training
McKeirnan & Hanson, 2023 [44]	Qualitative study	USA (Idaho)	Evaluate PTs’ opinions about administering immunizations	Administered vaccines	General immunizations	State-approved immunization training
Adams, 2023 [45]	Interventional (experimental)	USA (Idaho)	Examine impact of expanded PTs duties during COVID-19	Administered vaccines, conducted health testing	General immunizations	PREP Act training, state-specific regulations
DeMaagd & Pugh, 2023 [46]	Other (commentary)	USA	Explore the expanded role of pharmacy personnel in immunization practices	Some states allow PTs to administer vaccines under supervision	General immunizations	11+ training programs available for PTs vaccine administration
Fuschetto et al., 2023 [47]	Observational (cross-sectional)	USA (Ohio)	Evaluate perceptions of pharmacy technician-administered vaccines post-COVID-19	PTs administered vaccines under PREP Act	COVID-19, childhood vaccines	Federal PREP Act requirements: state licensure or national certification, ACPE training, CPR certification
McCormick et al., 2023 [48]	Qualitative study	USA	Analyze COVID-19 PBI service operations	Administered vaccines, assisted in workflow	COVID-19, routine vaccines	PREP Act training, state-specific requirements
Street & Taylor, 2023 [49]	Qualitative study	UK (England)	Define the role criteria for a ‘clinical’ pharmacy technician (CPT) in a Primary Care Network (PCN) setting	Future tasks: Administration of flu, COVID and Travel vaccination under a Patient Group Direction (PGD)	Flu and COVID-19 and travel (referenced under PGD)	Portfolio of competence; national frameworks; Level 5+ qualification; accredited training (e.g., CPPE pathways)
Miran et al., 2024 [50]	Observational (cohort)	USA	Evaluate impact of immunizing PTs on vaccination volume, workflow, and job satisfaction	Administered vaccines in community pharmacy chain	General immunizations	ACPE-approved training, CPR certification, state licensure/registration
Chadi et al., 2024 [51]	Qualitative study	Canada (Quebec)	Assess stakeholder perspectives on pharmacy vaccination	Assisted pharmacists in vaccine administration	General immunizations	Training and role delegation varied by pharmacy setting
Ayenew et al., 2024 [52]	Observational (cross-sectional)	Ethiopia	Assess readiness and barriers for pharmacy-led vaccination services	Expressed readiness, but faced regulatory and infrastructure challenges	General immunizations	Identified need for enhanced education and policy reform
Hursman et al., 2025 [53]	Interventional (pilot study)	USA (North Dakota)	Implement “VaxChamp” program for PTs vaccine advocacy	Advocated for vaccination, administered vaccines in some pharmacies	Pneumococcal, hepatitis B, shingles	Specialized training for advocacy, varied administration training
McKeirnan & Sarchet, 2025 [54]	Interventional	USA (Arizona)	Implement immunizing PTs in a federal healthcare facility	Administered vaccinations in a federal health system (Whiteriver Service Unit)	Various vaccines	Accredited immunization training from Washington State University

ACIP: Advisory Committee on Immunization Practices; ACPE: Accreditation Council for Pharmacy Education; CPR: Cardiopulmonary Resuscitation; DTP: Diphtheria, Tetanus, Pertussis; HepA: Hepatitis A; HepB: Hepatitis B; Hib: Haemophilus influenzae type b; HPV: Human Papillomavirus; MenACWY: Meningococcal conjugate vaccine (serogroups A, C, W, Y); MMR: Measles, Mumps, Rubella; PREP Act: Public Readiness and Emergency Preparedness Act; PBI: Pharmacy-based Immunization; PT(s): Pharmacy Technician(s); Tdap: Tetanus, Diphtheria, Pertussis (adult booster).

**Table 2:** Competencies and roles of pharmacy technicians in immunization services.

Intervention on Immunization Process Phase	Competency/Role	Description
Before	Inventory management	Manage vaccine storage, stock rotation and ordering [20,55].
Before	Patient screening support	Distribute and help with form filling, collecting patient information and identifying potential issues [18,20].
All phases (Before, During, After)	Work with other healthcare professionals	Work with local prescribers and healthcare departments to increase the size of vaccinated population and decrease the size of the public pool in which disease may reside [55].
All phases (Before, During, After)	Educate and communicate with the general public	Advocate for the appropriate use of vaccinations among the public [18].
During	Vaccine administration (in different countries/areas jurisdictions)	Administer vaccines (where legally allowed and with proper training) [18,20,22,29,36,44,54,55].
During, After	Monitor and report adverse reactions to vaccines	Know how to report any vaccine side effects to the responsible authority [56,57].
During, After	Sharp material handling and disposal	Safe preparation and disposal of sharp material, following accepted protocols (CDC’s Safe Injection Practices and FDA guidelines) and OSHA [18,55,58,59].
During, After	Keep immunization records	Accurately documenting immunization registries [55].

CDC: Centers for Disease Control and Prevention; FDA: Food and Drug Administration; OSHA: Occupational Safety and Health Administration.

## Data Availability

Requests to access the datasets should be directed to the corresponding author and will be granted upon reasonable request.

## Supplementary Materials

The following supporting information can be downloaded at: https://www.mdpi.com/article/10.3390/healthcare13151862/s1, Table S1: Immunization Administration Courses for Pharmacy Technicians: Components and Structure [80,81,82,83,84,85,86,87,88,89]; Table S2: Training Immunization Course Curricula; Supplementary File S1: Implementation Guidelines Toolkit.

## Abbreviations

The following abbreviations are used in this manuscript:

ACIP	Advisory Committee on Immunization Practices
ACPE	Accreditation Council for Pharmacy Education
APTUK	Association of Pharmacy Technicians UK
CDC	Centers for Disease Control and Prevention
CPR	Cardiopulmonary Resuscitation
DTP	Diphtheria, Tetanus, Pertussis
FDA	Food and Drug Administration
HepA	Hepatitis A
HepB	Hepatitis B
Hib	Haemophilus influenzae Type b
HPV	Human Papillomavirus
MeSH	Medical Subject Heading
MenACWY	Meningococcal Conjugate Vaccine (serogroups A, C, W, Y)
MMR	Measles, Mumps, Rubella
NS	Not Specified
OSHA	Occupational Safety and Health Administration
PBI	Pharmacy-based Immunization
PPE	Personal Protective Equipment
PREP Act	Public Readiness and Emergency Preparedness Act
PT	Pharmacy Technician
Tdap	Tetanus, Diphtheria, Pertussis (adult booster)
UK	United Kingdom
USA	United States of America
WHO	World Health Organization
