# Supplementary material for: Pharmacy Technicians in Immunization Services: Mapping Roles and Responsibilities Through a Scoping Review
Source: Healthcare (Basel). 2025 Jul 30;13(15):1862. doi: 10.3390/healthcare13151862 (PMC12346832; doi:10.3390/healthcare13151862)
Supplement: Supplementary file 1 [file healthcare-13-01862-s001.zip › healthcare13151862-Supplementary material.pdf]

healthcare
MDPI

# Pharmacy Technicians in immunization services: mapping roles and responsibilities through a scoping review

**Carolina Valeiro [1], Vítor Silva [2], Jorge Balteiro [3], Diane Patterson [4], Gilberto Bezerra [4], Karen Mealiff [4], Cristiano Matos [1], Ângelo Jesus [5] and João Joaquim [3,*]**

[1] European Association of Pharmacy Technicians, Brussels, Belgium; carolinavaleiro99@gmail.com (C.V.); cristiano.r.matos@gmail.com (C.M.)
[2] Unidade Local de Saúde de Coimbra, EPE, 3004-561 Coimbra, Portugal; vitorahsilva@gmail.com (V.S.)
[3] Escola Superior de Tecnologia da Saúde, Instituto Politécnico de Coimbra, Coimbra, Portugal; balteiro@estesc.ipc.pt
[4] Department of Pharmaceutical Sciences and Biotechnology, Technological University of the Shannon: Midlands Midwest, Dublin Road, Athlone, Ireland; diane.pa7erson@tus.ie (D.P.), gilberto.bezerra@tus.ie (G.B.), karen.mealiff@tus.ie (K.M.)
[5] LAQV/REQUIMTE, Escola Superior de Saúde, Instituto Politécnico do Porto, Porto, Portugal acj@ess.ipp.pt
* Correspondence: joaojosejoaquim@gmail.com

**Supplementary Materials:** The following supporting information is part of the paper "Pharmacy Technicians in Immunization Services: Mapping Roles and Responsibilities Through a Scoping Review" from Valeiro et al. (2025).

**Table S1.** Immunization Administration Courses for Pharmacy Technicians: Components and Structure.

**Table S2.** Training Immunization Course Curricula.

**Supplementary File S1.** Pharmacy Technician Immunization Course Plan: Implementation Guidelines Toolkit.

**Table S1.** Immunization Administration Courses for Pharmacy Technicians: Components and Structure.

| Feature/ Module | WHO Immunization training materials [1] | PTCB Immunization Administration Certificate [2] | CEimpact Immunization Administration Training for Pharmacy Technicians [3] | CB Training Vaccination, Basic Life Support and Anaphylaxis Training Course [4] | Administration of Vaccines and Injectable Medications [5] | Pharmacy Technician Online Training: Immunization [6] | Vaccinology Course [7] | PTU Elite: Immunizations [8] | Immunisation and Vaccination Training Course [9] | Vaccination Training [10] |
|---|---|---|---|---|---|---|---|---|---|---|
| Provider/ Organization | World Health Organization (WHO) | Pharmacy Technician Certification Board | CEimpact | CB Training | PLURAL | freeCE | East Africa Centre for Vaccines and Immunization | TRC Healthcare | University College Cork | Pharmaceutical Society of Ireland |
| Region | Global | United States | United States | UK | Portugal | United States | Uganda | United States | Ireland | Ireland |
| Course Duration | Self-paced | Total of 6 contact hours, combined with self-study and live components. | 6-hour course including 5 hours of self-study and 1-hour live virtual workshop. | 3.5 hours in-person practical workshops | 8 hours | 6.5-hours of self-paced online training | One week | 4-hour self-paced online study and 2 hours of supervised skill assessment | 3-hour | 2-hours |
| Format | Online, with sup. practical components recommended for in-person training (e.g.: supervised practice) | Online | Hybrid: Online self-study followed by a live virtual workshop. | In-person | Hybrid | Online | Hybrid: both virtual and in-person sessions held in Uganda. | Online | In-person | Online |

| Theoretical Modules | | | | | | | | | | |
|---|---|---|---|---|---|---|---|---|---|---|
| Roles of PT in supporting immunization | NS | ☑ | ☑ | NS | NS | ☑ | ☑ | ☑ | NS | NS |
| Immunization basic concepts | ☑ | ☑ | NS | NS | ☑ | ☑ | ☑ | ☑ | NS | NS |
| Anatomy and Physiology | NS | NS | ☑ | NS | NS | NS | NS | NS | ☑ | NS |
| Vaccine Pharmacology | ☑ | NS | NS | NS | NS | NS | ☑ | NS | NS | NS |
| Introduction to Public Health Concepts | ☑ | NS | NS | NS | NS | NS | NS | NS | NS | NS |
| Aseptic Concepts and Techniques | ☑ | NS | ☑ | NS | ☑ | NS | ☑ | ☑ | ☑ | NS |
| More common used vaccines and RA | ☑ | ☑ | ☑ | NS | ☑ | ☑ | ☑ | ☑ | ☑ | ☑ (Seasonal influenza, pneumococcal polysaccharide, herpes zoster and COVID-19) |
| Vaccine preventable diseases | ☑ | ☑ | ☑ | NS | ☑ | ☑ | ☑ | ☑ | ☑ | ☑ |

| | | | | | | | | | | |
|---|---|---|---|---|---|---|---|---|---|---|
| Vaccine Administration Technique | ☑ | ☑ | ☑ | NS | ☑ | ☑ | ☑ | ☑ | ☑ | ☑ |
| Material needed for vaccine administration | ☑ | NS | NS | NS | NS | NS | NS | ☑ | ☑ | NS |
| Anatomic Site and Route Selection | ☑ | ☑ | ☑ | NS | ☑ | ☑ | ☑ | ☑ | ☑ | NS |
| Immunization Schedules | ☑ | ☑ | ☑ | NS | ☑ | NS | ☑ | ☑ | ☑ | NS |
| Vaccine Handling, Storage and Disposal | ☑ | ☑ | ☑ | ☑ | ☑ | ☑ | ☑ | ☑ | ☑ | |
| Contraindications and adverse reactions | ☑ | ☑ | ☑ | ☑ (“Common complications” | ☑ | ☑ | ☑ | ☑ | ☑ | ☑ |
| Follow-up | ☑ | NS | NS | ☑ | NS | NS | NS | NS | ☑ | NS |
| Legal and ethical considerations | NS | ☑ | ☑ | ☑ | ☑ | ☑ | ☑ | ☑ | ☑ | ☑ |
| Actions in emergency situations | ☑ | ☑ | ☑ | ☑ (in children, adults, and infants) | ☑ | | ☑ | NS | ☑ | ☑ - separate 4-hour course “Responding to an Emergency Situation & |

| | | | | | | | | | | |
|---|---|---|---|---|---|---|---|---|---|---|
| | | | | | | | | | | Management of Anaphylaxis” |
| Vaccine Development Process | NS | NS | NS | NS | NS | NS | ☑ | NS | NS | NS |
| Communication skills | ☑ | NS | ☑ “Describe best practices for immunizing children and adolescents including site selection and distraction techniques” | NS | NS | ☑ “identify distraction techniques that can be used when administering immunizations” | ☑ | ☑ | ☑ | NS |
| Vaccination guidelines in specific groups (pediatric, pregnant women, immunocompromised) | ☑ | NS | ☑ | NS | ☑ | ☑ | ☑ | NS | ☑ | ☑ |
| Sharp Equipment Disposal | ☑ | NS | NS | NS | NS | NS | NS | NS | ☑ | NS |

 https://doi.org/10.3390/ healthcare13151862

| Practical Modules | | | | | | | | | | |
|---|---|---|---|---|---|---|---|---|---|---|
| Hands-on injection training | NS but recommended. | ✗ "Procedures for vaccine administration: SQ, IM, IN" only online | ☑ | ☑ | ☑ | ☑ - "You will then record your demonstration" | ☑ | ☑ | ☑ IM injection | ☑ (seasonal influenza. Pneum. polysaccharide, herpes zoster (shingles) and COVID-19 vaccines. |
| CPR or emergency response training | NS | ✗ "Managing vaccine-related adverse reactions and emergency situations" only online | ☑ | ☑ - includes CPR for adults, children, and infants, recovery position, and choking management | ☑ | NS | ☑ | NS | NS | ☑ - CPR course in adults and children |
| Supervised Clinical Practice | NS but recommended. | NS | ☑ | ☑ | ☑ | NS | ☑ | NS | NS | NS |
| Certification included | Yes. | Yes; requires passing the PTCB Immunization Administration Exam. | Yes; includes assessments and provides certificate upon completion. | NS | Yes; participants receive a participation cert. from PLURAL. Competency certificate is issued by "Ordem dos Farmacêuticos" | Yes; all free CE courses are accredited by the ACPE, meeting all state and national requirements. | Yes; each participant who attends more than 90% of sessions will earn CPD points and a certificate at the end of the course. | Yes; upon completion participants receive certification. | Yes; "Certificate Irish Nursing & Midwifery Board of Ireland in immunization & Vaccination" | Yes; upon successful completion, pharmacists are authorized to supply and administer specified vaccines for two years. |

 https://doi.org/10.3390/ healthcare13151862

Legend: ACPE = Accreditation Council for Pharmacy Education; CPD = Continuing Professional Development; CPR = Cardiopulmonary Resuscitation; IM = Intramuscular injection; IN = Intranasal administration; NS = Not Specified; SQ = Subcutaneous injection; WHO – World Health Organization.


1. WHO Immunization training materials. https://www.who.int/teams/immunization-vaccines-and-biologicals/essential-programme-on-immunization/training.
2. PTCB-Immunization Administration. https://www.ptcb.org/credentials/https%3A%2F%2Fwww.ptcb.org%2Fcredentials%2Fimmunization-administration-certificate%3F6830f614746b1.
3. Immunization Administration Training for Pharmacy Technicians - CEimpact. https://www.ceimpact.com/training/immunization-administration-training-for-pharmacy-technicians/.
4. Vaccination including Basic Life Support and Anaphylaxis Training. https://cb-training.com/vaccination-training/.
5. PLURAL- Administração de Vacinas e Injetáveis. https://www.plural.pt/formacao/administracao-de-vacinas-e-injetaveis-inicial-blearning/.
6. Pharmacy Technician Online Training: Immunization (2024). In: freeCE. https://www.freece.com/courses/immunization/.
7. Vaccinology Course – ECAVI. https://e-cavi.com/registration-open-for-10th-vaccinology-course/.
8. PTU Elite: Immunizations. In: TRC Healthc. https://trchealthcare.com/product/ptu-elite-immunizations/.
9. Immunisation & Vaccination Training. In: ER Train. https://www.ertraining.ie/immunisation-vaccination-training.
10. Vaccinations Training | PSI. https://www.psi.ie/education-and-training/vaccinations-training.

**Table S2.** Training Immunization Course Curricula.

**Pharmacy Technician Immunization Course Curricula**

**Curriculum Overview and Structure**

**Total Duration:** 8 hours.
**Format:** Blended (5 hours theory, 3 hours practical/simulation).
**Target Audience:** Pharmacy Technicians (Pre-service or In-service Training).

**Entry Requirement:** This course must be directed at qualified/certified pharmacy technicians who have completed a recognized professional education program in their countries.
Trainees must demonstrate prior knowledge in basic immunology, anatomy, physiology, pharmacology, public health, pharmacovigilance, and ethics.

**Delivery:** Onsite, hybrid, or online with mandatory practical sessions.

**Curriculum Map Summary**

- **Foundational Science** (Modules 1–2)
- **Public Health and Safety** (Modules 3–5)
- **Professional Practice and Clinical Skills** (Modules 6–8)

| **1.Immunology and Vaccine Science (1 hours)** | |
|---|---|
| **Module Overview** | This module introduces pharmacy technicians to the essential concepts of immunology, focusing on the immune system's role in vaccination and immunization. It will cover the basic mechanisms of the immune system, the different types of immunity, and the role of vaccines in disease prevention. This module aims to equip pharmacy technicians with the knowledge to understand immunological concepts critical for supporting immunization efforts and advising patients on vaccine-related topics. |
| **Learning Outcomes** | 1. Explain the basic concepts of immunology including the structure and function of the immune system.<br>2. Describe the different types of immunity (innate and adaptive immunity) and their roles in disease defense.<br>3. Identify the components of the immune system (e.g., white blood cells, antibodies, antigens, and cytokines) and their functions.<br>4. Understand the principles of vaccine development and the immunological basis of how vaccines work.<br>5. Describe the concept of herd immunity and its importance in public health.<br>6. Identify common vaccine-preventable diseases and their associated vaccines.<br>7. Discuss the safety and potential side effects of vaccines and the importance of monitoring patients' post-immunization.<br>8. Recognize the role of pharmacy technicians in the immunization process, including patient education and vaccine administration support. |
| **Indicative Syllabus (Theory)** | **Introduction to Immunology**<br>• Overview of the immune system.<br>• Primary and secondary immune responses.<br>• Components of the immune system: White blood cells, Antibodies and antigens, Cytokines and the inflammatory response.<br>• The importance of immunology in health and disease prevention.<br><br>**Types of Immunity**<br>• Innate and Adaptive immunity. |

<table>
<tr><td></td><td>• Active vs. Passive immunity.<br>Vaccine Basics: How Vaccines Work<br>• The immunological basis of vaccination: The concept of immunization and immune memory.<br>• Types of vaccines:<br>  o Live attenuated vacines.<br>  o Inactivated (killed) vacines.<br>  o Subunit, recombinant, conjugate vaccines.<br>  o mRNA vacines.<br>• Understanding adjuvants and their role in vaccine efficacy.<br>Herd Immunity and Its Role in Public Health<br>• Definition and significance of herd immunity.<br>• How herd immunity helps protect vulnerable populations.<br>• The relationship between vaccination rates and the spread of disease.<br>• The role of the pharmacy technician in supporting herd immunity through immunization programs.<br>Immunization Schedule and Vaccines<br>• Overview of common vaccines:<br>  o Childhood immunization schedule.<br>  o Adult vaccines.<br>  o Special vaccines (e.g., HPV, COVID-19).<br>• Vaccine indications, contraindications, and precautions.<br>Vaccine Safety and Side Effects<br>• Common side effects of vaccines.<br>• Rare side effects and adverse events.<br>• Monitoring and reporting adverse reactions.<br>• Vaccine contraindications and precautions.<br>• Ensuring patient safety through appropriate vaccine storage, handling, and administration.<br>The Role of Pharmacy Technicians in Immunization<br>• Educating patients about vaccines, side effects, and post-vaccination care.<br>• Managing vaccination records and documentation.<br>• Understanding legal and ethical considerations in immunization.</td></tr>
<tr><td>Teaching Method</td><td>• Theoretical instruction through asynchronous online learning and live virtual lectures.<br>• Case studies and problem-solving workshops.<br>• Interactive group discussions and reflective practice.</td></tr>
<tr><td>Evaluation Method</td><td>• Complete a multiple-choice quiz assessing understanding of immune responses, vaccine classifications, and key immunological concepts.<br>• Answer short-answer or case-based questions to explain active versus passive immunity and differentiate between various vaccine types.</td></tr>
</table>

 https://doi.org/10.3390/ healthcare13151862

| **2. Anatomy and Physiology (1 hours)** | |
|---|---|
| **Module Overview** | This module introduces the structure and function of the human body, with a focus on the physiological processes that relate to the immune system and immunization. Understanding basic human anatomy and physiology will help a better understanding of the effects of vaccines and how they interact with the body. Emphasis will be placed on the systems that directly support immune function and vaccination, including the lymphatic system, circulatory system, and the nervous system. |
| **Learning Outcomes** | 1. Identify and describe the main structures of the human body relevant to immunology, such as the immune system, lymphatic system, and circulatory system.<br>2. Explain the key physiological processes that underpin the immune response, including antigen recognition, inflammation, and immune memory.<br>3. Describe the physiological effects of vaccines on the body, including how the immune system responds to vaccination.<br>4. Understand the role of major organs and tissues in immune function, such as the spleen, bone marrow, thymus, and lymph nodes.<br>5. Explain how the circulatory system supports immune function by transporting immune cells and antibodies.<br>6. Discuss the relationship between anatomy and physiology in the context of immunization and vaccine response.<br>7. Recognize the signs and symptoms of common vaccine reactions based on physiological processes.<br>8. Understand the pharmacokinetics of vaccines, including their absorption, distribution, and elimination in the body. |
| **Indicative Syllabus (Theory)** | **Introduction to Human Anatomy and Physiology**<br>• Overview of human body systems.<br>• Key concepts: Homeostasis, cellular functions, and organ systems.<br>**The Immune System and Its Anatomy**<br>• Primary and secondary lymphoid organs.<br>• The lymphatic system role in fluid balance and immune response.<br>**The Circulatory System and Its Role in Immunity**<br>• Blood composition and the circulatory route.<br>• The role of the heart and blood vessels in maintaining circulation of immune components.<br>• The importance of the circulatory system in vaccine delivery.<br><br>**Inflammation and Immune Response**<br>• Acute vs. chronic inflammation: Physiological basis and immune response.<br>• The role of cytokines, chemokines, and other inflammatory mediators.<br>• Phagocytosis.<br>• The role of antibodies in the immune response.<br>• Immune memory: Formation of memory cells following vaccination and natural infection. |
| **Teaching Method** | • Theoretical instruction through asynchronous online learning and live virtual lectures.<br>• Case studies and problem-solving workshops. |
| **Evaluation Method** | • Complete multiple-choice and diagram-labeling questions on anatomical sites for vaccine administration and related physiological structures.<br>• Perform practical identification of correct injection sites using diagrams, mannequins, or anatomical models. |

<table>
<tr><th colspan="2">3. Public Health, Vaccine Policy and Pharmacovigilance (1 hours)</th></tr>
<tr><td>Module Overview</td><td>This module introduces the principles of public health, the importance of vaccination in preventing disease, and the role of pharmacovigilance in ensuring the safety and efficacy of vaccines. Students will gain an understanding of public health strategies for vaccination, the role of healthcare professionals in immunization campaigns, and the process of monitoring vaccine safety through pharmacovigilance systems. The module will emphasize the importance of reporting adverse events and maintaining patient safety.</td></tr>
<tr><td>Learning Outcomes</td><td>1. Understand the key principles of public health, the role of immunization in public health and how vaccines contribute to disease prevention and control.<br>2. Describe the process of pharmacovigilance and its significance in monitoring vaccine safety and efficacy.<br>3. Identify common vaccine adverse effects and distinguish between normal and abnormal reactions.<br>4. Understand the systems for reporting adverse drug reactions and the role of pharmacy technicians in pharmacovigilance.<br>5. Describe vaccine policy frameworks and international strategies.<br>6. Discuss the role of the pharmacy technician in supporting public health efforts, including patient education, vaccine administration, and data reporting.<br>7. Recognize the importance of vaccine safety monitoring and how this impacts patient care and public health outcomes.</td></tr>
<tr><td>Indicative Syllabus (Theory)</td><td>Introduction to Public Health and Vaccination<br>• Overview of Public Health: Definition, goals, and the role of public health in disease prevention.<br>• Immunization as a public health tool: History of vaccination and its impact on global health.<br>• Global and national vaccination strategies: The role of the World Health Organization (WHO) and national health authorities in immunization efforts.<br>The Role of Pharmacy Technicians in Public Health<br>• Pharmacy technicians' role in immunization programs:<br>  o Supporting vaccination efforts in pharmacies, clinics, and healthcare settings.<br>  o Educating patients on the benefits of immunization and addressing vaccine concerns.<br>  o Ensuring proper vaccine storage, handling, and record-keeping.<br>• Public health education: Communicating the importance of vaccination to different population groups.<br>Introduction to Pharmacovigilance<br>• Definition and purpose of pharmacovigilance.<br>• Pharmacovigilance systems and reporting.<br>• Reporting mechanisms: How to report adverse drug reactions (ADRs) and vaccine side effects.<br>• Ethical and Legal Aspects of pharmacovigilance.<br>Vaccine Safety: Side Effects and Adverse Events<br>• Common side effects of vaccines: Local and systemic reactions.<br>• Causality and severity.<br>• Monitoring for adverse reactions and follow-up.<br>The Role of Pharmacy Technicians in Pharmacovigilance<br>• Recognizing and reporting adverse events: Understanding when and how to report vaccine-related adverse events.<br>• Role of pharmacy technicians in preventing, identifying, and mitigating risks.</td></tr>
<tr><td>Teaching Method</td><td>• Theoretical instruction through asynchronous online learning and live virtual lectures.<br>• Interactive group discussions and reflective practice.<br>• Case studies and problem-solving workshops.</td></tr>
<tr><td>Evaluation Method</td><td>• Complete multiple-choice or matching questions on pharmacovigilance procedures, common vaccine side effects, and monitoring protocols.</td></tr>
</table>

<table>
<tr><th colspan="2">4. Vaccine - Specific Knowledge and Schedules (0,5 hours)</th></tr>
<tr><td>Module Overview</td><td>This module provides an in-depth understanding of various aspects of vaccines, including their routes of administration, commonly used vaccines, vaccine-preventable diseases, vaccination schedules, and the proper procedures for storing, handling, and disposing of vaccines. The goal is to equip students with the knowledge needed to support vaccination efforts, ensure safe administration, and handle vaccines according to regulatory and safety guidelines.</td></tr>
<tr><td>Learning Outcomes</td><td>1. Identify and explain the different routes of vaccine administration and the rationale behind each.<br>2. Recognize the most used vaccines in various immunization schedules.<br>3. Describe common vaccine-preventable diseases, their symptoms, and associated vaccines.<br>4. Understand and interpret vaccine schedules, including childhood, adolescent, and adult immunization recommendations.<br>5. Demonstrate proper techniques for vaccine storage, handling, and disposal, ensuring compliance with legal and safety standards.</td></tr>
<tr><td>Indicative Syllabus (Theory)</td><td>Routes of Vaccine Administration<br>• Intramuscular (IM) injection: Common sites and vaccine types administered via IM.<br>• Subcutaneous (SC) injection: Common sites and vaccines administered via SC.<br>• Oral vaccines: Types of vaccines administered orally.<br>• Intranasal vaccines: Overview of intranasal administration.<br>• Intradermal vaccines: Mechanism and uses.<br>• Rationale for choosing administration routes: Factors influencing the route.<br>Commonly Used Vaccines<br>• Childhood Vaccines: DTP, IP, MMR, Hib, Hepatitis B and Rotavirus.<br>• Adult Vaccines: Influenza, Shingles (Zoster), Pneumococcal, Tdap, HPV.<br>• Other Vaccines: COVID-19 vaccines, Meningococcal vaccines, and Travel vaccines.<br>Vaccine-Preventable Diseases<br>• Impact of vaccines on global disease reduction (understand the concept of mortality and morbidity).<br>• Overview of vaccine effectiveness.<br>Vaccine Schedules<br>• Vaccine schedules by age range.<br>• Vaccine schedules for special populations: Pregnant women, immunocompromised individuals, travelers.<br>Vaccine Storage, Handling, and Disposal<br>• Vaccine storage:<br>o Refrigeration and freezing requirements.<br>o Monitoring temperature during storage and transportation.<br>• Handling vaccines:<br>o Preventing contamination.<br>o Preparing vaccines for administration (e.g., reconstitution of live vaccines).<br>o Best practices for using vaccine vials and syringes.<br>• Disposal of vaccine materials:<br>o Safe disposal of needles, syringes, and vaccine vials.<br>o Handling of expired or unused vaccines according to regulations.<br>o Environmental considerations and legal requirements for disposal.<br>Safety Practices in Vaccine Administration<br>• Patient screening.<br>• Proper technique for injections, managing side effects.<br>• Post-vaccination monitoring: Immediate reactions and follow-up.<br>• Documentation: Recording vaccination details, lot numbers, and adverse events.</td></tr>
<tr><td>Teaching Method</td><td>• Theoretical instruction through asynchronous online learning and live virtual lectures.<br>• Case studies and problem-solving workshops.</td></tr>
<tr><td>Evaluation Method</td><td>• Complete multiple-choice or matching questions on vaccine types, cold chain principles, recommended storage temperatures, and handling procedures.</td></tr>
</table>

https://doi.org/10.3390/ healthcare13151862

<table>
<tr><th colspan="2">5. Clinical Safety and Emergency Preparedness (1 hours)</th></tr>
<tr><td>Module Overview</td><td>This module provides the knowledge and skills needed to ensure patient safety during immunization. It covers the identification of vaccine contraindications, the management of adverse reactions, and the critical steps in emergency response.</td></tr>
<tr><td>Learning Outcomes</td><td>1. Identify and assess contraindications and precautions for common vaccines.<br>2. Develop the skills necessary to respond to adverse reactions promptly and effectively.<br>3. Implement emergency response procedures.<br>4. Communicate effectively with patients about potential risks and safety measures.<br>5. Document and report adverse reactions to the appropriate health authorities.<br>6. Understand the legal and ethical responsibilities related to vaccine safety and patient care.</td></tr>
<tr><td>Indicative Syllabus (Theory)</td><td>Vaccine Contraindications and Precautions<br>• Common contraindications: Allergic reactions, immunocompromised patients, pregnancy, acute illness or moderate to severe infection.<br>• Precautions: Conditions that may increase the risk of adverse reactions.<br>Understanding Adverse Reactions<br>• Types of Adverse Reactions: Local, systemic and allergic reactions.<br>• Serious adverse reactions: criteria and examples.<br>Management of Adverse Reactions<br>• Immediate response to adverse reactions:<br>o Recognizing early signs of anaphylaxis and other severe reactions.<br>• Reporting adverse events:<br>o How and when to report adverse events to pharmacovigilance systems.<br>o Understanding the impact of accurate reporting on public health safety.<br>Emergency Response Concepts<br>• Recognizing and treating anaphylaxis:<br>o Symptoms.<br>o Using an epinephrine auto-injector (e.g., EpiPen) or pre-filled syringe.<br>o Dosage and timing for epinephrine administration.<br>• Basic life support (BLS) principles:<br>o Airway management, breathing support, circulation, and defibrillation.<br>• Emergency kit.<br>Legal and Ethical Responsibilities in Vaccine Safety<br>• Legal obligations: Reporting adverse reactions to national systems and understanding state and federal regulations regarding adverse event reporting.<br>• Ethical considerations: Informed consent, patient autonomy, and confidentiality.</td></tr>
<tr><td>Teaching Method</td><td>• Role-playing and patient communication simulations.<br>• Emergency drills and scenario-based response training.</td></tr>
<tr><td>Evaluation Method</td><td>• Complete a quiz or case study assessing clinical decision-making in vaccine administration.</td></tr>
</table>

 https://doi.org/10.3390/ healthcare13151862

| **6. Professional Role Development and Ethics (0,5 hours)** | |
|---|---|
| **Module Overview** | This module highlights the evolving responsibilities of PTs as vital members of the healthcare team, contributing to patient safety, vaccination access, and health education. It also addresses professional ethics, ongoing professional development, and the regulatory framework that governs PT practice in immunization. |
| **Learning Outcomes** | 1. Describe the evolving role of pharmacy technicians in immunization services.<br>2. Understand the scope of practice for PTs in vaccine administration and support.<br>3. Identify the critical tasks performed by PTs in the immunization process.<br>4. Communicate effectively with patients about vaccines, including addressing hesitancy and providing accurate information.<br>5. Demonstrate professionalism and ethical decision-making in the context of immunization.<br>6. Understand the regulatory and legal requirements for PTs involved in vaccine administration.<br>7. Participate in professional development to enhance immunization skills and knowledge. |
| **Indicative Syllabus (Theory)** | **The Evolving Role of Pharmacy Technicians in Immunization**<br>• Historical perspective on the PT role in pharmacy practice.<br>• Growth of PT responsibilities in response to public health needs.<br>**Scope of Practice for PTs in Immunization**<br>• Core responsibilities:<br>o Patient screening for contraindications.<br>o Vaccine preparation and drawing doses.<br>o Assisting with vaccine administration.<br>o Documentation and record-keeping.<br>**Critical Tasks in the Immunization Process**<br>• Vaccine storage and handling: Ensuring cold chain integrity and proper inventory management.<br>• Vaccine preparation: Reconstitution, dose measurement, and injection site identification.<br>• Patient interaction: Screening patients, addressing vaccine-related concerns and providing post-vaccination care instructions.<br>• Documentation: Recording vaccine administration details.<br>**Communication Skills in Immunization**<br>• Fundamentals of effective communication: Verbal and non-verbal communication and Building patient trust and rapport.<br>• Informed consent in immunization.<br>• Managing vaccine hesitancy: Handling challenging conversation.<br>**Professionalism and Ethical Practice**<br>• Ethical principles in healthcare.<br>• Professional behavior in high-stress situations.<br>**Legal and Regulatory Considerations**<br>• Understanding the legal scope of PT practice in immunization.<br>• State and national regulations. |
| **Teaching Method** | • Interactive group discussions and reflective practice.<br>• Role-playing and patient communication simulations. |
| **Evaluation Method** | • Complete multiple-choice mini-test on regulations and scope of practice in immunization. |

| **7. Practical Training and Infection Control (3 hours)** | |
|---|---|
| **Module Overview** | This integrated module provides pharmacy technicians with supervised, hands-on practice in vaccine administration, along with essential training in personal safety and infection control. It covers the practical skills needed for accurate injection techniques, proper use of personal protective equipment (PPE), and maintaining a safe working environment. |
| **Learning Outcomes** | 1. Demonstrate proper injection techniques for different routes of vaccine administration.<br>2. Safely and effectively handle needles, syringes, and other vaccination equipment.<br>3. Apply best practices for infection control and personal safety during immunization.<br>4. Properly use and dispose of personal protective equipment to reduce the risk of cross-contamination and exposure.<br>5. Understand the importance of hygiene and workspace cleanliness in a clinical setting.<br>6. Maintain a safe and organized immunization station. |
| **Indicative Syllabus (Theory)** | **Injection Techniques and Hands-On Practice**<br>• Understanding different routes of administration: Intramuscular, Subcutaneous, Intradermal.<br>• Correct needle selection and angle for each route.<br>• Proper handling and preparation of vaccines.<br>• Aseptic technique and minimizing contamination risk.<br>• Simulated practice with training models and supervised patient encounters.<br>• Assessing injection site for suitability and patient comfort.<br>• Managing needle stick injuries and bloodborne pathogen exposure.<br>**Personal Protective Equipment and Infection Control**<br>• Types of PPE in immunization settings: Gloves, masks, face shields, gowns, eye protection.<br>• Proper donning and doffing procedures.<br>• Hand hygiene and the role of alcohol-based sanitizers.<br>• Preventing cross-contamination between patients.<br>• Disposal of sharp and biohazardous waste.<br>**Maintaining a Safe Immunization Environment**<br>• Organizing and maintaining a clean and safe vaccination station.<br>• Handling spills and potential contamination incidents.<br>• Routine disinfection of high-touch surfaces and equipment. |
| **Indicative Syllabus (Practical)** | **Practical Exercises and Simulations**<br>• Hands-on practice with injection models.<br>• Role-playing patient interactions and consent discussions.<br>• Emergency drills for managing needle stick injuries.<br>• Peer assessments and instructor feedback on technique. |
| **Teaching Method** | • Supervised practice in vaccine preparation and administration.<br>• Emergency drills and scenario-based response training. |
| **Evaluation Method** | • Conduct supervised demonstrations of vaccine preparation, administration, emergency response, and documentation, with direct observation and feedback. |

On completion of the course, the trainee will be able to independently and safely plan, perform, and document vaccination procedures in compliance with current legal, ethical, and clinical standards, while effectively communicating with patients to promote health literacy and contribute to improved vaccination coverage in the population.

 https://doi.org/10.3390/ healthcare13151862

**Supplementary File S1.** Pharmacy Technician Immunization Course Plan: Implementation Guidelines Toolkit.

**Pharmacy Technician Immunization Course Plan**
**Implementation Guidelines Toolkit**

This toolkit provides structured guidelines to support the formal integration of Pharmacy Technicians into immunization services. It provides recommendations for the implementation of a standardized recommended immunization training program, with the goal of ensuring educational consistency, safeguarding patient safety, and promoting cross-border recognition of competencies across Europe.

**1. Target Audience and Entry Requirements**

This course is intended for qualified/certified Pharmacy Technicians who have completed a recognized professional education program in their countries.

Trainees should have a relevant background and demonstrate prior knowledge in basic immunology, anatomy, physiology, pharmacology, public health, pharmacovigilance, and ethics.

**2. Curriculum Structure and Content**

The curriculum must be modular and competency-based, with theoretical components delivered onsite, online, or in a hybrid format. Practical sessions are mandatory and must be conducted in person to ensure the development of hands-on skills and professional competencies.

- Theoretical modules must include:
    - Fundamentals of immunization (e.g., immune response, herd immunity);
    - Common vaccine-preventable diseases and their epidemiology;
    - Vaccine schedules and guidelines by age/risk group;
    - Injection techniques and site selection;
    - Vaccine storage, handling, and disposal (e.g., cold chain);
    - Legal and ethical responsibilities;
    - Patient education and communication;
    - Public health framework and the Pharmacy Technician's role.

- Practical modules must include:
    - Supervised practice of intramuscular and subcutaneous injection techniques;
    - Emergency response training, including cardiopulmonary resuscitation (CPR) and management of adverse events;
    - Documentation and pharmacovigilance (e.g., adverse event reporting systems);

**3. Evaluation and Certification**

Trainees must undergo continuous assessment during the course, including quizzes, simulations, and observed practice.

It is recommended that a final written and practical examination be included to assess both theoretical understanding and technical competencies.

A minimum passing score is recommended, with at least 70% suggested as a benchmark for successful completion. Certification must be awarded after successful completion of all modules and assessments.

The certification should be valid for a defined period (e.g., three to five years), with renewal dependent on the following criteria:

- Proof of continued practice (e.g., minimum number of administered vaccines in a certain period of time);
- Completion of a refresher module with updated practical guidelines and clinical content;
- Reassessment or validation of skills if required by national standards.

**4. Practical training**

Practical training should be supervised by qualified healthcare professionals trained in immunization practices (e.g., pharmacists, nurses, doctors, or pharmacy technicians) or by certified trainers with appropriate immunization expertise. All trainers must be appropriately accredited, possess relevant professional experience, and demonstrate specific expertise in immunization practices.

Training environments must be equipped with appropriate, safe, and accessible facilities and materials necessary for both theoretical and practical components.

Quality assurance system must be in place to continuously monitor the quality of course delivery, assess learning outcomes, and incorporate feedback through regular evaluation and review processes.

**5. Integration with Professional Frameworks and National Contexts**

Legal and regulatory frameworks can differ across countries. In some countries, Pharmacy Technicians are not currently authorized to administer vaccines. These political and regulatory barriers must be addressed case by case, considering:

- National legislation for immunization;
- Professional body recommendations;
- Public health needs;
- Existing workforce shortages.

To address these barriers effectively, a combination of strategies should be considered, including:

- Stakeholder engagement and policy advocacy at national level
- Pilot programs to demonstrate feasibility and safety
- National dialogue guided by international best practices and public health imperatives

**6. Recognition**

The training program could be included in the National and European Continuing Professional Development (CPD) systems for Pharmacy Technicians. Professional organizations, Health authorities and regulators could collaborate to:

- Recognize this training as part of professional career advancement and development;
- Define the scope of practice aligned with public health priorities;
- Establish a registry of trained and certified Pharmacy Technicians immunizers/vaccinators.

Flexibility should be built into the course design to allow for language translation and modular customization, enabling adaptation to local healthcare systems, legal requirements, and population health needs, while maintaining adherence to agreed core competencies and learning outcomes.

To further support consistency and comparability, it is recommended to explore the development of a future European registry where certified training programs and qualified Pharmacy Technicians can be registered.

**7. Sustainability, Access, and Expansion**

Training should be scalable, with options for low-cost delivery, particularly in underserved or rural areas.

Public-private partnerships, EU-funded programs, and national health budgets can be leveraged to ensure widespread implementation.

The model should be periodically reviewed and updated to reflect:

- New vaccine technologies
- Evolving public health threats
- Legal changes
- Feedback from trainers, Pharmacy Technicians, and health authorities

**8. Life-Saving Response Skills – CPR and Emergency Training**

Our recommendation is that Immunization training for Pharmacy Technicians should be embedded within or linked to Basic Life Support and/or First Aid training, enabling Pharmacy Technicians to manage emergency scenarios during and after administration.

The main objectives and outcomes for those training is described below:

**Main objectives:**

- Equip participants with fundamental knowledge of emergency response principles.
- Develop practical skills in CPR (Cardiopulmonary Resuscitation) for adults, children, and infants.
- Enable effective use of an AED (Automated External Defibrillator).
- Train for immediate action in choking, bleeding, shock, burns, and other medical emergencies.
- Promote confidence and preparedness in handling real-life emergencies situations safely.

**Main Learning Outcomes:**

- By the end of the course, participants will be able to:
- Demonstrate proper CPR techniques for different age groups.
- Safely and effectively use an AED in emergency scenarios.
- Recognize signs and symptoms of common medical emergencies.
- Perform basic first aid (e.g., control bleeding, treat burns, manage shock).
- Apply the chain of survival and understand its importance.
- Respond calmly and effectively while awaiting emergency services.
